# Spatial expression of claudin 18.2 in matched primaries and metastases of tubo-ovarian carcinoma of all subtypes

**DOI:** 10.1007/s00428-024-03756-1

**Published:** 2024-02-07

**Authors:** Paul Wagner, Paul Gass, Patrik Pöschke, Markus Eckstein, Laura Gloßner, Arndt Hartmann, Matthias Wilhelm Beckmann, Peter Andreas Fasching, Matthias Ruebner, Julius Emons, Ramona Erber

**Affiliations:** 1 Institute of Pathology, University Hospital Erlangen, Friedrich-Alexander-Universität Erlangen-Nürnberg, Comprehensive Cancer Center Erlangen-EMN (CCC ER-EMN), Erlangen, Germany; 2 Department of Gynecology and Obstetrics, University Hospital Erlangen, Friedrich-Alexander-Universität Erlangen-Nürnberg, Comprehensive Cancer Center Erlangen-EMN (CCC ER-EMN), Erlangen, Germany

**Keywords:** Tubo-ovarian cancer, Mucinous tubo-ovarian carcinoma, CLDN18.2, Immunohistochemistry, Targeted therapy

## Abstract

Physiologically, claudin 18 splice variant 2 (CLDN18.2) expression is restricted to the gastric epithelium, but its expression has been detected in solid cancers. Zolbetuximab, a chimeric IgG1 antibody targeting CLDN18.2, has demonstrated promising effects in patients suffering from CLDN18.2-positive, HER2-negative locally advanced gastric cancer and is currently being studied further. To date, little is known about CLDN18.2 expression in other histological subtypes of tubo-ovarian carcinoma (TOC) and their matching metastases.

Using a cohort of all histological TOC subtypes, we investigated the immunohistochemical (IHC) CLDN18.2 expression in both TOCs (*n* = 536), their matching metastatic tissue (*n* = 385) and in 93 metastases without primary. Tissue microarrays comprised both the tumor center and periphery. IHC positivity was defined as biomarker expression of ≥ 75% in tumor cells with moderate-to-strong membranous staining.

Overall CLDN18.2 positivity was 4.1% (21/515) in the TOC centers and 3.6% (18/498) in their peripheries. In primaries of mucinous tubo-ovarian carcinoma (MTOC), CLDN18.2 positivity rates were 45% (18/40) and 36.6% (15/41), respectively. Positivity rates for the corresponding metastases were 33% (4/12, center) and 27% (3/11, periphery). The expression was relatively homogenous throughout all tumor sites. With no expression in 99.5% of nonmucinous tumors, CLDN18.2 positivity was almost exclusively seen in the mucinous subtype.

In tubo-ovarian carcinoma, CLDN18.2 expression was, with rare exceptions, restricted to the mucinous subtype. Among them, 33% of metastasized MTOCs presented with CLDN18.2 positivity. Hence, CLDN18.2 might display a promising target for personalized therapy in patients with advanced MTOC.

**Supplementary Information:**

The online version contains supplementary material available at 10.1007/s00428-024-03756-1.

## Introduction

With 313,959 diagnoses and 207,252 deaths worldwide in 2020, tubo-ovarian carcinoma (TOC) is the eighth most common cancer in terms of incidence and mortality in women [1]. However, the disease has the highest case fatality rate among all gynecologic cancers, with a 5-year overall survival of only 50% across all stages [2]. Although there have been improvements in survival for women with TOC in recent years, the majority of cases is still diagnosed in FIGO stage III or IV [3]. While the advent of poly (ADP-ribose) polymerase (PARP) inhibitors has significantly expanded the therapeutic options for advanced high-grade serous and endometrioid carcinomas, especially for those being *BRCA*-deficient, their effect does not extend to all histological subtypes of TOC [4, 5].

For a group of cancers with rather unfavorable prognoses, including gastric, esophageal and pancreatic carcinoma, a promising biomarker has emerged [6]. Introduced by Sahin et al. in 2008, claudin 18.2 (CLDN 18.2) is the second splice variant of claudin 18, a member of the heterogeneous claudin protein family, which comprises at least 26 types in humans [7]. Physiologically, its expression is restricted to differentiated gastric epithelia and is not yet detectable in gastric stem cells [8]. Claudins are crucially involved in the formation of tight junctions (TJs) and are therefore responsible for the polarity, barrier and transcellular transport function of epithelia [9]. Aiming for CLDN18.2 as a potential therapeutic target seems attractive for several reasons. First, systemic interactions of drugs addressing CLDN18.2 seem quite unlikely through its exclusivity in the described tissue and some cancers. At the same time, due to the loss of polarity and dislocation to the cell surface, CLDN.18.2 becomes more accessible to antibodies in malignant tissue [10]. Zolbetuximab, a chimeric IgG1 antibody targeting CLDN18.2, has been developed for this purpose [11]. In clinical phase I and II studies, it has been shown to significantly prolong progression-free (PFS) and overall survival (OS) in patients suffering from CLDN18.2-positive, HER2-negative locally advanced gastric cancer [12–14]. According to the first published results of two phase III studies, it seems to hold its substantial promise in clinical study cohorts [15, 16]. Aside from gastric and esophageal carcinomas, Zolbetuximab is currently also being studied in other advanced solid cancers in clinical trials [17].

Even though a relevant expression of CLDN18.2 was described early in a subset of ovarian cancers as well, thorough research in this field has not been pursued [8]. Similar to clinical trials, previous immunohistochemical studies have focused on the impact of CLDN18.2 as a prognostic and therapeutic biomarker in gastric cancer [18, 19]. In terms of TOC, there is only one investigation to the best of our knowledge comparing CLDN18.2 expression in mucinous tubo-ovarian carcinomas (MTOCs) with metastatic gastrointestinal mucinous carcinoma (MGMC). Interestingly, both MGMCs from the upper gastrointestinal tract and MTOCs were reported to express CLDN18.2 in up to 84% of cases, while filiae from the lower gastrointestinal tract appeared entirely negative [20]. Apart from this postulated use of excluding metastases from the lower gastrointestinal tract as a differential diagnosis, little is known about the potential diagnostic value of CLDN18.2 in ovarian cancer in general.

We therefore aimed to elucidate this lack of knowledge by performing a large-scale immunohistochemical analysis across the entire spectrum of TOC subtypes and matching peritoneal spread tissue and distant metastases.

## Materials and methods

### Patient cohort

**Fig. 1:**
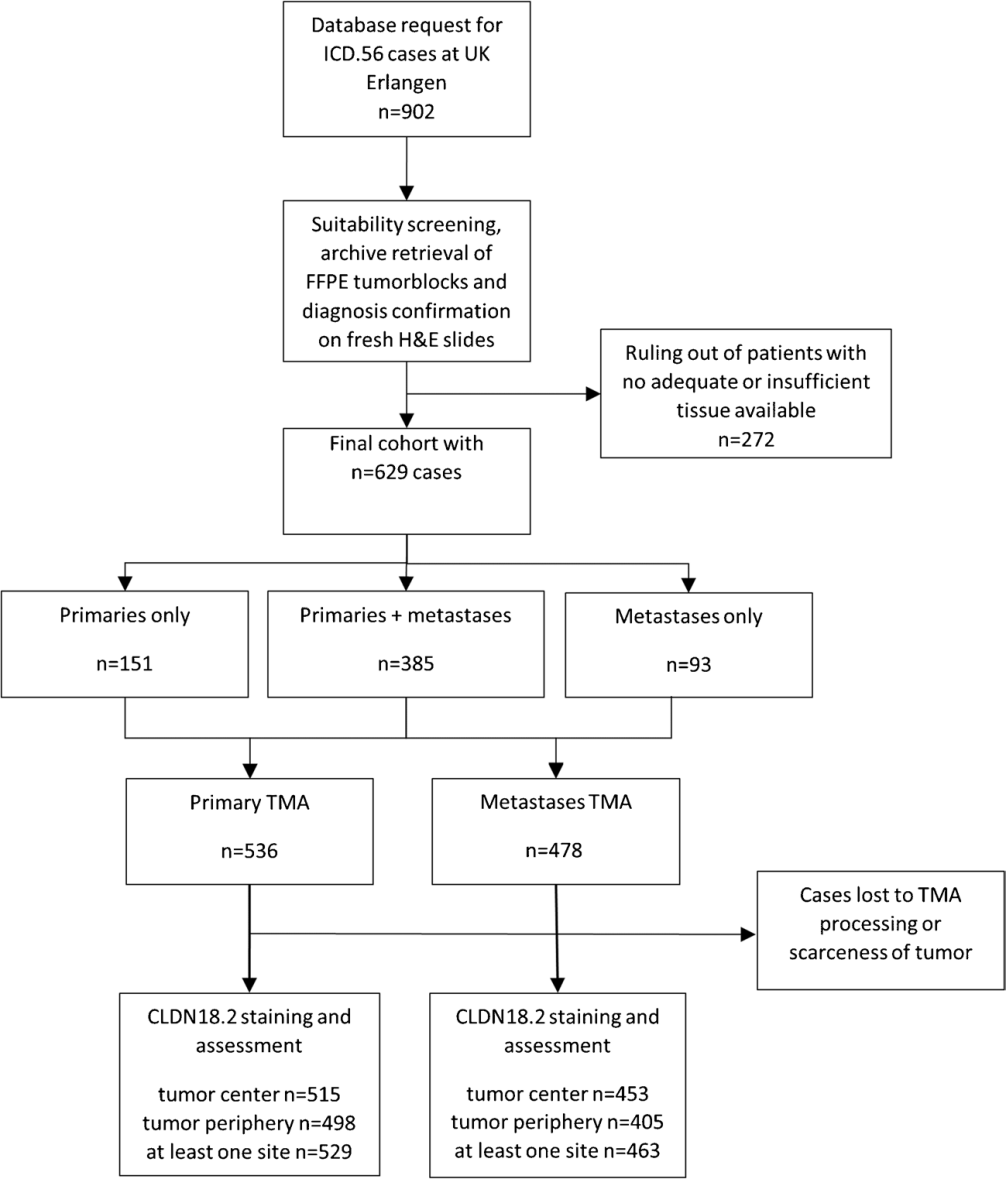
Consort diagram illustrating the composition of the study cohort (Abbreviations: ICD, International Statistical Classification of Diseases and Related Health Problems; FFPE, formalin-fixed paraffin-embedded; H&E, hematoxylin and eosin; TMA, tissue microarray)

All cases of TOC diagnosed and treated at the University Hospital Erlangen (UKER) between 2003 and 2021 included in the Bavarian Clinical Cancer Registry were screened for their suitability for the study. All patients underwent surgical resection of primary and/or secondary lesions. Availability of tissue in domo, sufficient tumor amount and distinct malignancy were taken as inclusion criteria for our tissue microarray (TMA) cohort. Cases with borderline tumors of epithelial origin were excluded. The formalin-fixed, paraffin-embedded (FFPE) TOC tissue was selected on the basis of medical records and retrieved from the archives of the Institute of Pathology UKER. Fresh hematoxylin and eosin (H&E) slides were fabricated from each tumor block. Taking the initial pathologic diagnosis into account, every case was reevaluated by a board-certified pathologist (R.E.). Histological subtypes were categorized according to the 5th edition of the WHO Classification of Tumors [21]. FIGO and TNM staging was performed according to the current versions of UICC TNM and FIGO classification for the entire cohort [22, 23]. Cases prior to 2014 staged with previous classifications were reclassified within their main category where applicable [24, 25]. Uncertain diagnoses (e.g., high-grade cancer, origin not assessable, or ovarian cancer, not otherwise specified) were additionally confirmed or excluded by immunohistochemistry. The final cohort consisted of 629 patients in total, containing all histological subtypes of TOC. If available, both cancer tissue from the ovary as well as cancer spread from, e.g., the peritoneum, lymph nodes or infiltrated organs, was collected. For the latter group, the term metastases will be frequently employed in the following, which includes both distant metastases (M1; e.g., liver metastasis) and cancer infiltrates disconnected from the main tumor mass in the ovaries (i.e., higher T stages) [23]. Eventually, sufficient FFPE tissue of *n* = 536 TOCs, *n* = 385 matching tubo-ovarian carcinoma metastases (TOCM) and *n* = 93 metastases without primary were included. After processing, cutting and staining the TMAs, *n* = 529 primary and *n* = 463 cancer spread samples were evaluable. For *n* = 619 patients, at least one tumor core was assessable. Figure 1 depicts the workflow from the cohort assembly until the final IHC assessment.

### TMA construction and claudin 18.2 immunohistochemistry

After the final cohort was gathered, randomization in terms of histologic subtype and year of first diagnosis was performed to prevent analytical biases. In the resulting order, the cases were ascendingly allocated to the TMA slots. Next, the areas of interest were marked on the H&E slides. Correspondent punch cores of 1.5 mm in diameter were taken manually from the donor paraffin blocks and transferred to the TMA recipient blocks. Primaries and metastases were split into separate TMAs. To investigate the intratumoral heterogeneity of CLDN18.2 expression, separate TMAs for both the tumor center and periphery were constructed.

Immunohistochemical staining was conducted on 1–2-µm thick sections of formalin-fixed paraffin embedded (FFPE) TMA tumor blocks on the Ventana BenchMark Ultra automated system (Ventana Medical Systems, Inc., Tucson, AZ, USA) after the institute’s standards and the manufacturers recommended protocol. For the CLDN18.2 antibody, a primary anti-human murine monoclonal antibody (clone: 43-14A; host: mouse, isotype: IgG1; Ventana Medical Systems, Inc., Tucson, AZ, USA) was used. Deparaffinization, pretreatment and antigen unmasking with CC1 at 100 °C for 64 min were followed by antibody incubation at 37 °C for 32 min. The binding of the antibody to CLDN18.2 was visualized with the optiView DAB IHC Detection Kit (Ventana). Eventually, subsequent counterstaining with hematoxylin and Bluing Reagent (Ventana) was conducted. For on-slide positive controls and the establishment of the antibody, human gastric mucosal tissue was employed.

### IHC scoring

After review of morphology in the TMA H&Es IHC, assessment was performed by an experienced gynecopathologist (R.E.) and a resident trained in gynecopathology (P.W.) blinded to the clinical data. Decision making was consensus based and on the basis of the phase III SPOTLIGHT biomarker study criteria [15]. The assessment was done manually with a Zeiss Axio microscope (magnification of × 50, × 100, × 200, and × 400). The percentage of positive tumor cells and the staining intensity were evaluated with regard to only the cell membrane. The percentage of stained tumor was visually estimated in 5% increments. Staining intensity was measured semiquantitatively and divided into four categories (0 as no staining, 1 as weak, 2 as moderate, and 3 as strong). CLDN18.2 positivity was defined as biomarker expression of ≥ 75% in tumor cells with moderate-to-strong membranous staining [15]. To quantify the whole staining pattern, the IRS score after Remmele and Stegner, taking staining percentage and intensity into account, was also calculated [26]. A minimum of 100 viable tumor cells was required for assessment.

### Statistical analysis

Statistical analysis was performed with GraphPad PRISM version 8 (GraphPad Software, Inc., San Diego, CA, USA). Paired *t* tests, Wilcoxon tests and Mann‒Whitney *U* tests were used for the comparison of intra- and intertumoral matched pairs and expansile vs. infiltrative subtypes, respectively. Varying expression patterns between subtypes were analyzed with the Kruskal‒Wallis test. A *p* value < 0.05 was considered significant.

## Results

### Characteristics of the study cohort

The histological subtype distribution in the final cohort was as follows: high-grade serous carcinoma *n* = 445, low-grade serous carcinoma *n* = 40, endometrioid carcinoma *n* = 43, mucinous carcinoma *n* = 43, clear-cell carcinoma *n* = 36, carcinosarcoma *n* = 17, malignant Brenner tumor *n* = 4, and undifferentiated, not otherwise specified *n* = 1. Table 1 gives clinicopathological data about the cohort. Of the total of *n* = 478 metastases, *n* = 384 were located in the peritoneal cavity, *n* = 17 were regional lymph node metastases and *n* = 58 originated from the parenchyma of pelvic or intraabdominal organs. *N* = 37 metastatic samples were from relapses. According to the 8th edition of the UICC TNM classification for TOC, *n* = 19 were distant metastases [23]. More detailed information about the numbers, localizations and size of the metastatic tissue are provided in the Supplementary Table S1.

**Table 1:** Clinicopathologic data of the patient cohort

	All tubo-ovarian carcinomas	High-grade serous	Low-grade serous	Endo-metrioid	Mucinous	Clear cell	Carcino-sarcoma	Others
Total *n* =	629 (100%)	445 (70.7%)	40 (6.4%)	43 (6.8%)	43 (6.8%)	36 (5.7%)	17 (2.7%)	5 (0.8%)
Age, mean ± SD range	60.98 ± 13.24 (19–95)	62.47 ± 12.21 (24–95)	52.71 ± 17.70 (19–85)	58.07 ± 13.05 (35–84)	55.39 ± 14.67 (19–83)	59.66 ± 14.00 (32–88)	64.52 ± 8.22 (50–81)	65.27 ± 18.94 (44–84)
FIGO stage								
I	124	43	9	22	29	18	1	2
II	43	32	5	3	1	1	0	1
III	398	314	26	17	10	14	15	2
IV	64	56	0	1	3	3	1	0
pT status								
T1	140	56	9	24	29	18	2	2
T2	59	45	6	4	1	2	0	1
T3	427	341	25	15	13	16	15	2
unknown	3	3						
pN status								
N0	251	147	19	33	23	22	5	2
N1	230	196	10	5	3	6	9	1
NX	148	102	11	5	17	8	3	2
Tumor grade								
Low grade		0	40	24	34	0	0	0
High grade		445	0	19	9	36	17	5

### Overall CLDN18.2 expression

**Fig. 2:**
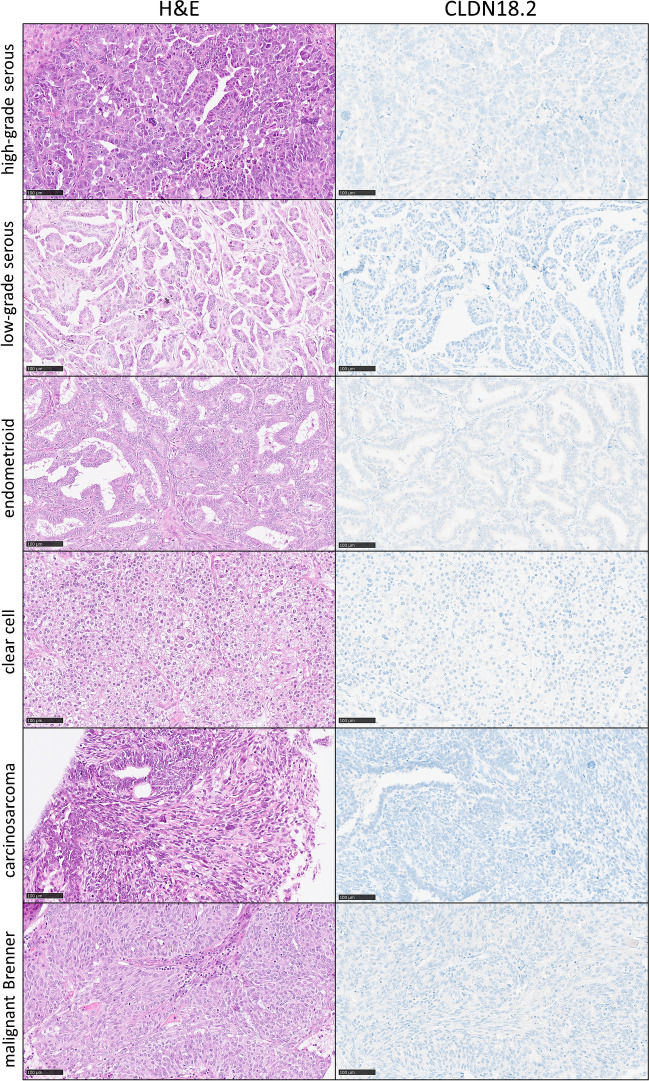
Typical H&E morphology and immunohistochemical CLDN18.2 staining patterns of non-mucinous tubo-ovarian carcinoma subtypes (magnification × 200, bar = 200 µm)

**Fig. 3:**
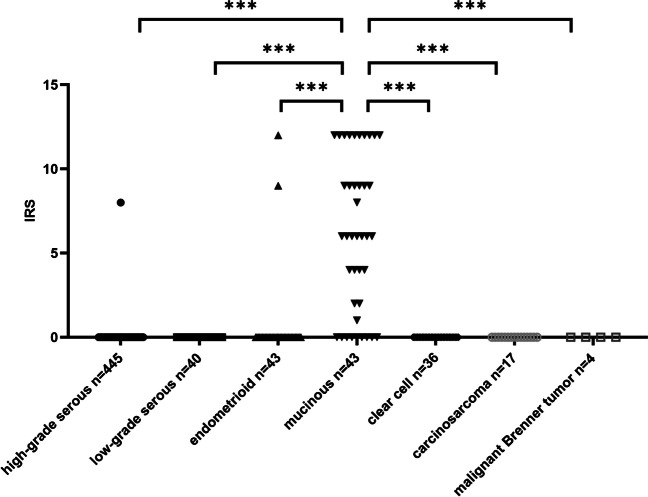
CLDN18.2 expression in the tumor center of primaries (indicated by IRS score) across subtypes (*** indicates a high significance with *p* value < 0,0001; Abbreviations: IRS, immunoreactive score). Apart from 1 serous and 2 endometrioid carcinomas, only mucinous carcinomas showed expression of CLDN18.2

From overall *n* = 619 evaluable patients, a total of *n* = 44 cases (7.1%) showed membranous CLDN 18.2 staining to some extent. The vast majority of cases (*n* = 575) was entirely CLDN18.2 negative. Figure 2 illustrates the typical appearance of all nonmucinous TOC subtypes in CLDN18.2 immunohistochemistry. Applying the SPOTLIGHT III criteria, CLDN 18.2 was positive in *n* = 28 cases (4.5%) in at least one of the tumor sites [15]. In regard to primaries, CLDN18.2 was detected in 4.1% (21/515) of TOC centers and in 3.6% (18/498) of TOC peripheries. In metastases, the positivity quota was only 0.9% (4/453) in the center and 0.7% (3/405) in the periphery. Apart from 1 out of 445 high-grade serous carcinomas and 2 out of 43 endometrioid carcinomas, CLDN18.2 was highly significantly restricted to MTOCs (Fig. 3).

### Intratumoral CLDN18.2 expression in primary mucinous carcinomas

**Fig. 4:**
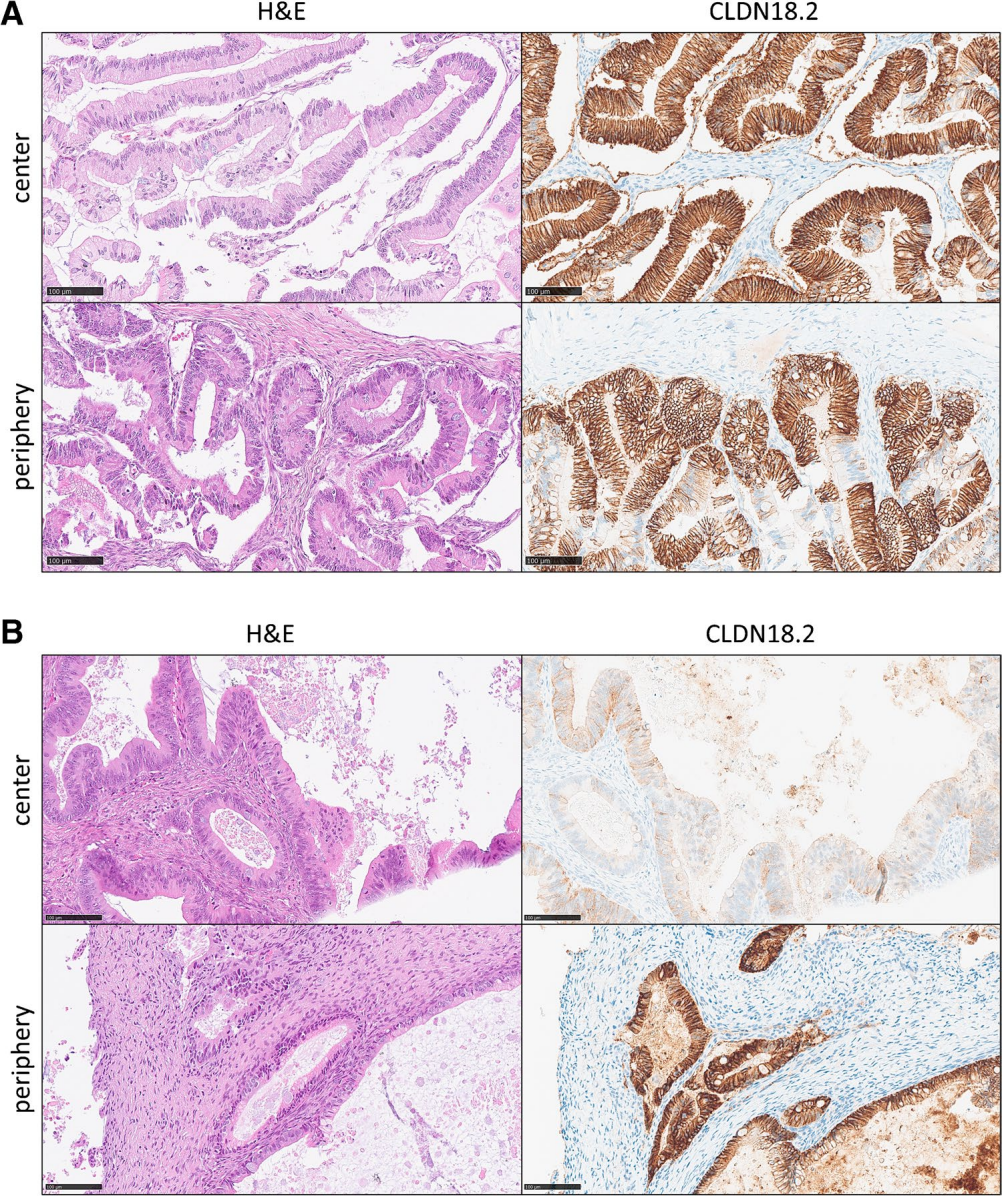
Examples of intratumoral matched pairs with (**A**) illustrating a concordant pair with positive expression in both the tumor center and periphery and (**B**) depicting a couple that shows discordant positivity in the periphery but weak expression in the center (magnification × 200, bar = 200 µm; Abbreviations: H&E, hematoxylin & eosin; IHC, immunohistochemistry)

Among MTOCs, however, CLDN18.2 positivity rates were 45% (18/40) in the tumor centers and 36.6% (15/41) in their peripheries. The mean IRS score in primaries was 6.125 (SD 4.57; 95%-CI: 4.67–7.59) in the center and 6.81 (SD 4.41; 95%-CI: 5.41–8.2) in the periphery. Paired *t* test did not reveal a significant difference in IRS scores between the two sites (*p* value = 0.1881). Overall intratumoral concordance, meaning that the matched pairs of center and periphery were both negative or positive with respect to the SPOTLIGHT III criteria, was 76.9% (30/39) (Supplementary Figure S1). Figure 4 displays the central and peripheral CLDN 18.2 expression of selected MTOC cases.

### Intratumoral CLDN18.2 expression in metastatic mucinous carcinomas

Positivity rates for the corresponding mucinous tubo-ovarian carcinoma metastases (MTOCMs) were 31% (4/13, center) and 23% (3/13, periphery). In the central area, the mean IRS score was 5.54 (SD 4.74; 95% CI: 2.68–8.4) compared to 5.08 (SD: 3.59; 95% CI: 2.91–7.25) in the peripheral area. As in primaries, the IRS score did not differ significantly between the localizations (*p* value = 0.6563). 92% (12/13) of the MTOCM pairs were intratumorally concordant (Supplementary Figure S2).

### Intertumoral CLDN18.2 expression in primary and metastatic mucinous carcinomas

**Fig. 5:**
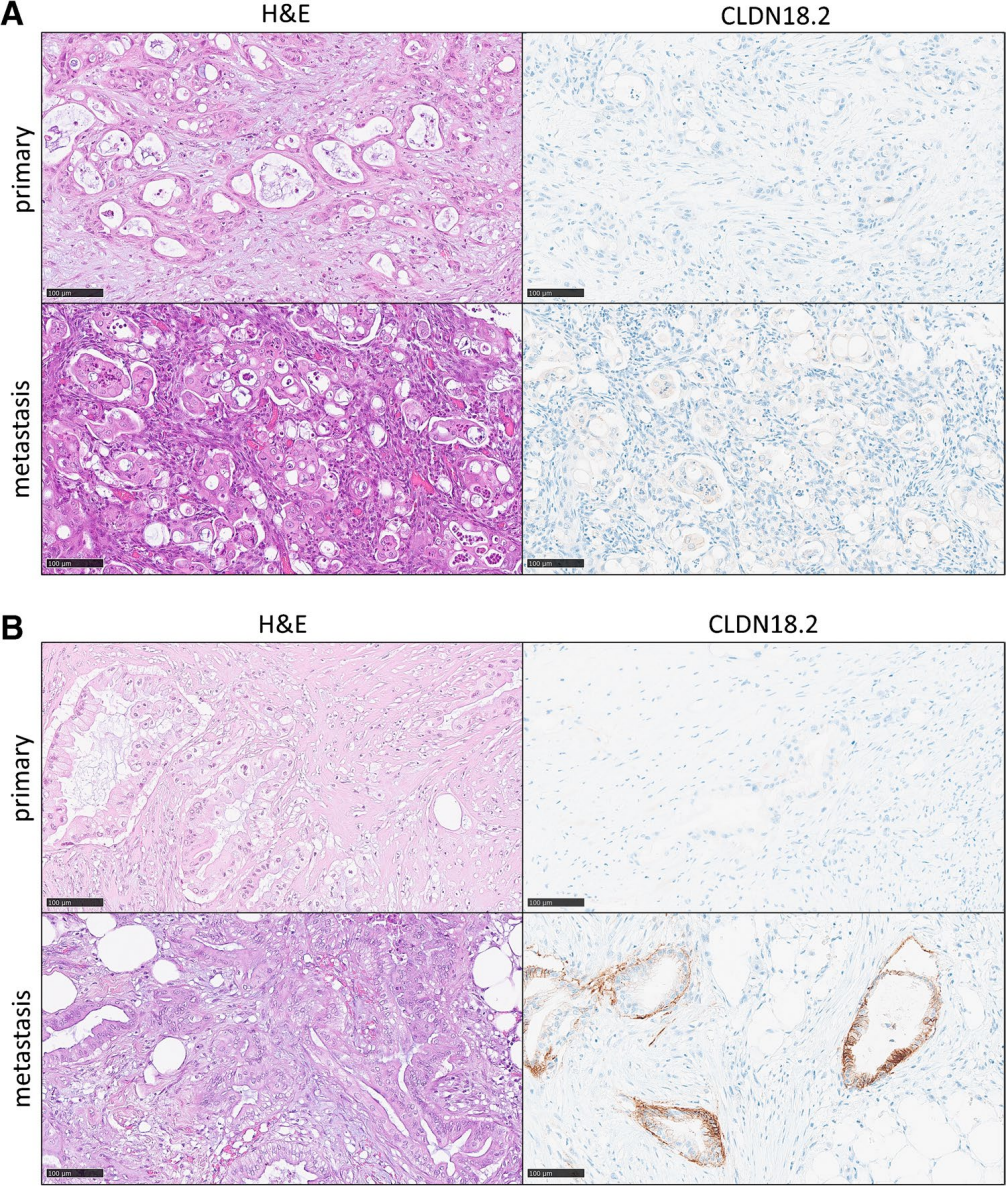
Examples of intertumoral matched pairs where (**A**) shows a case in which CLDN18.2 is concordantly negative at both tumor sites and (**B**) illustrates a couple, where CLDN18.2 is discordantly negative in the ovary but positive in the metastasis (magnification × 200, bar = 200 µm; Abbreviations: H&E, hematoxylin & eosin; IHC, immunohistochemistry)

A total of *n* = 12 pairs of primaries and corresponding metastases of MTOC could be matched. The couples were considered concordant in their CLDN18.2 expression if positivity was granted for both localizations in at least one of the tumor areas (center or periphery). Thus, 58% (7/12) of the couples were concordant, whereas 42% (5/12) were discordant by definition. However, the mean IRS scores in the center and periphery of primaries and metastases did not vary significantly (*p* value (center) = 0.3594, *p* value (periphery) = 0.1875) (Supplementary Figure S3). Figure 5 shows examples of concordant and discordant combinations of primary and metastatic lesions.

### Expansile vs infiltrative mucinous carcinoma

The current WHO classification 5th edition no longer divides mucinous carcinomas into three grades but distinguishes between an expansile and infiltrative growth pattern, which has been shown to better correlate with prognosis [27]. Expansile growth refers to extensive back-to-back gland proliferation with preserved differentiation and is associated with lower stage, reduced invasiveness and better prognosis [28, 29]. Of the 43 cases in our cohort, 31 showed an expansile growth pattern, and 12 showed an infiltrative growth pattern. We observed CLDN 18.2 expression to be emphasized in the expansile subtype, which shows a high similarity to gastrointestinal epithelium. The expression then tends to decrease during the process of dedifferentiation toward infiltrative growth but is nonetheless preserved to a noteworthy extent in this type. This is reflected by a positivity rate of 58% (18/31) in expansile and 42% (5/12) in infiltrative mucinous ovarian carcinomas. Their mean IRS scores did not differ significantly (*p* value (center) = 0.2842, *p* value (periphery) = 0.1085). Supplementary Figures S4 and S5 provide case examples of the staining behavior of the two subtypes in both primaries and metastases.

## Discussion

The aim of the present study was to obtain an overview of the overall status of the anecdotally reported CLDN18.2 prevalence in tubo-ovarian carcinoma across all histological subtypes and tumor sites. In order to estimate CLDN18.2’s potential to cover an unmet need for targeted therapy in TOC, this differentiated consideration of its expression seems an important prerequisite.

### CLDN18.2 is highly specific for mucinous carcinoma

In summary, we were able to robustly support the previous scientific findings. Our results are consistent with the few preexisting data that have linked CLDN18.2 expression exclusively to the mucinous subtype of TOC. The overall CLDN18.2 positivity of 4.5% (*n* = 28/619), allows the studies’ first conclusion that CLDN18.2 will supposedly not display a general target for personalized therapy in patients with TOC to be drawn quickly. In the direct comparison, CLDN18.2 was more frequent in primaries (*n* = 24/529, 4.5%) than in metastases (*n* = 4/463, 0.9%), which could supposedly be explained by a comparably higher percentage of high-grade serous carcinoma among the metastases.

### The value of CLDN18.2 in differential diagnosis

Simultaneously, this stringent restriction to the mucinous subtype offers opportunities from a diagnostic point of view. For example, the differential diagnosis between endometrioid and mucinous carcinomas can be challenging, since both subtypes can mimic the morphologic features of one another. On the other hand, we noticed a slight emphasis of CLDN18.2 in gastrointestinal differentiated MTOCs. Although the classification of mucinous borderline tumors in intestinal (IBMT) and endocervical types (EBMT) has in the meantime been abandoned, a study in 2013 reported a similar pattern for borderline tumors. They postulated a Mullerian origin of EBMT, which explains the absence of CLDN18.2 as a marker of gastric lineage [30]. Our findings point in the same direction, leaving behind questions about the exact definition and origin of mucinous carcinomas, which are still subject to scientific debate [31, 32]. Another unsolved problem in this context has always been the difficult distinction between MTOCs and metastatic lesions from the gastrointestinal tract. Recalling the results of Wang et al., CLDN18.2 can help differentiate metastases from the upper gastrointestinal tract (CLDN18.2 +) from those of the lower gastrointestinal tract (CLDN18.2 −) [20]. Unfortunately, it cannot cover the demand for a much-needed marker to reliably distinguish MTOC from metastatic mucinous carcinomas, a decision which, in case of doubt, has to be made on the basis of clinical and imaging criteria.

### Mucinous carcinoma is a distinct subtype of tubo-ovarian carcinoma

The mucinous subtype is exceptionally rare, accounting for only 3% of ovarian carcinomas [33]. At the same time, MTOCs differ distinctly from other subtypes in their biological behavior. Compared to the dominant high-grade serous carcinoma, they tend to be diagnosed at an earlier stage, while the affected women are usually younger, with one-quarter of the patients being under 44 years of age [34]. Meanwhile, mucinous carcinoma is classically characterized by a bad response to platinum-based chemotherapy, leading to a very poor outcome in the case of advanced disease [35]. The two previous studies performing CLDN18.2 IHC in TOC raised with 22 or 17 cases about half of the patients, respectively [8, 20]. In addition, their study objectives differed from ours, and only mucinous carcinomas were included. Wang et al. reported a positivity rate of 86.4% in TOC, which at first glance differs greatly from our observations. However, they defined a cutoff value of 50% of the tumor cells stained with moderate intensity to assign cases positive. Transferring this scoring system to our cohort would change the positive percentage from 46 to 56%. Under the condition of moderate staining (2 +) in at least 60% of the cells, Sahin et al. reported a positivity rate of 24%. As their findings widely spread from one another, our study contributes additional information to estimate the actual CLDN18.2 frequency in MTOC. Since data from the phase II study of Tureci et al. suggest a superior efficacy of Zolbetuximab in patients with CLDN18.2 expression in ≥ 75% of the tumor mass, a rather high cutoff value seems appropriate [36].

### Intra- and intertumoral distribution of CLDN18.2 expression in mucinous carcinoma

Overall, we were unable to find a strong emphasis of CLDN 18.2 expression at a particular tumor site. As the tumor periphery and metastases are thought to be the relevant scenes of invasion, the question about CLDN18.2’s role in tumor progression and dissemination remains unanswered. Interestingly, claudin subtypes 3 and 4 have been particularly linked to the invasive character of TOC [37]. A gene analysis found them to be among the most upregulated genes in TOC cells [38]. Knockdown of *CLDN3* or *CLDN4* in tumor cell lines results in reduced tight junction formation and increased invasiveness [39]. Several immunohistochemical studies could associate high expression levels of *CLDN3* and *CLDN4* with poorer survival [40–42]. These findings support the hypothesis that claudin-mediated alterations in tight junction integrity with subsequent loss of polarity and cellular adhesion represent hallmarks of carcinogenesis [43].

### Targeted therapy of CLDN18.2

The recently published results of the SPOTLIGHT and GLOW studies promote large expectations [15, 16]. In a large set of locally advanced or metastasized gastric cancers, a regimen of Zolbetuximab + mFOLFOX6 (5-fluoruracil plus oxaliplatin and leucovorin) showed significant superiority compared to the placebo-controlled group. The risk of progression or death was reduced by 25%, and the median PFS was prolonged by approximately 2 months. Median overall survival rose from 15.54 to 18.23 months, resulting in a hazard ratio concerning the risk of death of 0.75 [15]. These outcomes raise the hope of potentially being transferable to other CLDN18.2-positive entities, such as MTOCs. Its pronounced expression in the predominantly gastrointestinal differentiated MTOCs illustrates the close biological relationship of the two entities that might come along with a similar vulnerability toward targeted therapy. As our study provides evidence for the occurrence of CLDN18.2 at all tumor sites, a therapy with, e.g., Zolbetuximab might be able to temper the current therapeutic deficit in advanced MTOC. However, in view of the epidemiology of the disease, realistic expectations should be maintained. MTOCs, which are very rare anyway, are usually diagnosed at an early stage and can then be adequately treated surgically. Finding a sufficient number of patients for clinical trials has already proven to be complicated and the potential target group for an anti-CLDN18.2 therapy may be too small to arouse the serious interest of trial sponsors [44].

### Limitations

The restriction to immunohistochemical analyses only is obviously a limitation of this study. For a deeper understanding of *CLDN18.2* gene expression in TOC, concomitant mRNA analyses would be desirable. On the other hand, with high availability and rather low cost, IHC as a diagnostic means in practical use has several advantages. Furthermore, it proves the expression of markers on a protein level. Beyond that, companion diagnostic tests for therapeutic antibodies, which in the case of CLDN18.2 already exist, are usually based on IHC [45]. Another point of criticism to mention is the relatively small statistical power of our analysis. Despite the long retrospective timeframe, only 43 cases of MTOC could eventually be assessed. Since mucinous carcinomas, compared to other subtypes of TOC, have a rather small tendency to spread metastatically, the metastases also did not reach a statistically robust number. Nonetheless, the approach succeeded in revealing certain tendencies in expression patterns and contributing additional insights to the yet scarce information on the topic.

## Conclusion

By including both the entire histologic variety of TOC as well as primaries and metastases split into subregions, our study delivers a global image of CLDN18.2 expression in ovarian cancer. We were able to immunohistochemically confirm CLDN18.2 as a biomarker highly specific for and common in all shapes of MTOC. Here, it seems to be a marker strongly related to gastrointestinal differentiation, appearing to be emphasized in low grade and thus expansile growing tumors. Nonetheless, the expression is to a relevant extent conserved in dedifferentiated infiltrative and metastasizing carcinomas, which are clinically the most complicated to handle. Future scientific efforts should try to correlate these findings with RNA and gene expression data. As for CLDN18.2 diagnostic and therapeutic means are already established, it could with comparably low effort be evaluated as a potential target in advanced mucinous tubo-ovarian carcinoma. Despite the low number of cases, these results raise the question of whether feasible clinical studies assessing the efficacy and tolerability of anti-claudin agents in MTOC patients positively tested for CLDN18.2 would nevertheless be worthwile.

### Supplementary Information

Below is the link to the electronic supplementary material.

Supplementary file1 (PDF 2513 KB)

## References

[1] Sung H, Ferlay J, Siegel RL, Laversanne M, Soerjomataram I, Jemal A, Bray F (2021) Global Cancer Statistics 2020: GLOBOCAN estimates of incidence and mortality worldwide for 36 cancers in 185 countries. CA Cancer J Clin 71:209–249. 10.3322/caac.21660 33538338 10.3322/caac.21660

[2] American Cancer Society (2023) Survival rates for ovarian cancer. https://www.cancer.org/cancer/types/ovarian-cancer/detection-diagnosis-staging/survival-rates.html. Accessed January 3, 2024

[3] Siegel RL, Miller KD, Fuchs HE (2022) Jemal A (2022) Cancer statistics. CA Cancer J Clin 72:7–33. 10.3322/caac.21708 35020204 10.3322/caac.21708

[4] González-Martín A, Pothuri B, Vergote I, DePont CR, Graybill W, Mirza MR, McCormick C, Lorusso D, Hoskins P, Freyer G, Baumann K, Jardon K, Redondo A, Moore RG, Vulsteke C, O’Cearbhaill RE, Lund B, Backes F, Barretina-Ginesta P, Haggerty AF, Rubio-Pérez MJ, Shahin MS, Mangili G, Bradley WH, Bruchim I, Sun K, Malinowska IA, Li Y, Gupta D, Monk BJ (2019) Niraparib in patients with newly diagnosed advanced ovarian cancer. N Engl J Med 381:2391–2402. 10.1056/NEJMoa1910962 31562799 10.1056/NEJMoa1910962

[5] Moore K, Colombo N, Scambia G, Kim BG, Oaknin A, Friedlander M, Lisyanskaya A, Floquet A, Leary A, Sonke GS, Gourley C, Banerjee S, Oza A, González-Martín A, Aghajanian C, Bradley W, Mathews C, Liu J, Lowe ES, Bloomfield R, DiSilvestro P (2018) Maintenance Olaparib in patients with newly diagnosed advanced ovarian cancer. N Engl J Med 379:2495–2505. 10.1056/NEJMoa1810858 30345884 10.1056/NEJMoa1810858

[6] Cao W, Xing H, Li Y, Tian W, Song Y, Jiang Z, Yu J (2022) Claudin18.2 is a novel molecular biomarker for tumor-targeted immunotherapy. Biomark Res 10:38. 10.1186/s40364-022-00385-1 35642043 10.1186/s40364-022-00385-1 PMC9153115

[7] Günzel D, Yu AS (2013) Claudins and the modulation of tight junction permeability. Physiol Rev 93:525–569. 10.1152/physrev.00019.2012 23589827 10.1152/physrev.00019.2012 PMC3768107

[8] Sahin U, Koslowski M, Dhaene K, Usener D, Brandenburg G, Seitz G, Huber C, Türeci O (2008) Claudin-18 splice variant 2 is a pan-cancer target suitable for therapeutic antibody development. Clin Cancer Res 14:7624–7634. 10.1158/1078-0432.Ccr-08-1547 19047087 10.1158/1078-0432.Ccr-08-1547

[9] Tsukita S, Furuse M, Itoh M (2001) Multifunctional strands in tight junctions. Nat Rev Mol Cell Biol 2:285–293. 10.1038/35067088 11283726 10.1038/35067088

[10] Li J (2021) Targeting claudins in cancer: diagnosis, prognosis and therapy. Am J Cancer Res 11:3406–3424 34354852 PMC8332862

[11] Mitnacht-Kraus R, Kreuzberg M, Utsch M, Sahin U, Türeci Ö (2017) Preclinical characterization of IMAB362 for the treatment of gastric carcinoma. Annals Oncol 28:v126. 10.1093/annonc/mdx367.012 10.1093/annonc/mdx367.012

[12] Sahin U, Tureci O, Manikhas G, Lordick F, Rusyn A, Vynnychenko I, Dudov A, Bazin I, Bondarenko I, Melichar B, Dhaene K, Wiechen K, Huber C, Maurus D, Arozullah A, Park JW, Schuler M, Al-Batran SE (2021) FAST: a randomised phase II study of zolbetuximab (IMAB362) plus EOX versus EOX alone for first-line treatment of advanced CLDN18.2-positive gastric and gastro-oesophageal adenocarcinoma. Ann Oncol 32:609–619. 10.1016/j.annonc.2021.02.005 33610734 10.1016/j.annonc.2021.02.005

[13] Türeci O, Sahin U, Schulze-Bergkamen H, Zvirbule Z, Lordick F, Koeberle D, Thuss-Patience P, Ettrich T, Arnold D, Bassermann F, Al-Batran SE, Wiechen K, Dhaene K, Maurus D, Gold M, Huber C, Krivoshik A, Arozullah A, Park JW, Schuler M (2019) A multicentre, phase IIa study of zolbetuximab as a single agent in patients with recurrent or refractory advanced adenocarcinoma of the stomach or lower oesophagus: the MONO study. Annals Oncol 30:1487–1495. 10.1093/annonc/mdz199 10.1093/annonc/mdz199 PMC6771222 31240302

[14] Sahin U, Schuler M, Richly H, Bauer S, Krilova A, Dechow T, Jerling M, Utsch M, Rohde C, Dhaene K, Huber C, Tureci O (2018) A phase I dose-escalation study of IMAB362 (Zolbetuximab) in patients with advanced gastric and gastro-oesophageal junction cancer. Eur J Cancer 100:17–26. 10.1016/j.ejca.2018.05.007 29936063 10.1016/j.ejca.2018.05.007

[15] Shitara K, Lordick F, Bang Y-J, Enzinger P, Ilson D, Shah MA, Van Cutsem E, Xu R-H, Aprile G, Xu J, Chao J, Pazo-Cid R, Kang Y-K, Yang J, Moran D, Bhattacharya P, Arozullah A, Park JW, Oh M, Ajani JA Zolbetuximab plus mFOLFOX6 in patients with CLDN18.2-positive, HER2-negative, untreated, locally advanced unresectable or metastatic gastric or gastro-oesophageal junction adenocarcinoma (SPOTLIGHT): a multicentre, randomised, double-blind, phase 3 trial The Lancet. 10.1016/S0140-6736(23)00620-7 10.1016/S0140-6736(23)00620-7 37068504

[16] Shah MA, Shitara K, Ajani JA, Bang Y-J, Enzinger P, Ilson D, Lordick F, Van Cutsem E, Gallego Plazas J, Huang J, Shen L, Oh SC, Sunpaweravong P, Soo Hoo HF, Turk HM, Oh M, Park JW, Moran D, Bhattacharya P, Arozullah A, Xu R-H (2023) Zolbetuximab plus CAPOX in CLDN18.2-positive gastric or gastroesophageal junction adenocarcinoma: the randomized, phase 3 GLOW trial. Nat Me 29:2133–2141. 10.1038/s41591-023-02465-7 10.1038/s41591-023-02465-7 PMC10427418 37524953

[17] ClinicalTrials.gov (2022) A study to assess the efficacy and safety of IMAB362 in combination with Nab-Paclitaxel and Gemcitabine (Nab-P + GEM) as first line treatment in subjects with claudin 18.2 (CLDN18.2) Positive, Metastatic Pancreatic Adenocarcinoma. https://clinicaltrials.gov/ct2/show/NCT03816163. Accessed April 5, 2023

[18] Arnold A, Daum S, von Winterfeld M, Berg E, Hummel M, Rau B, Stein U, Treese C (2020) Prognostic impact of Claudin 18.2 in gastric and esophageal adenocarcinomas. Clin Translat Oncol 22:2357–2363. 10.1007/s12094-020-02380-0 10.1007/s12094-020-02380-0 PMC7577914 32488802

[19] Dottermusch M, Krüger S, Behrens HM, Halske C, Röcken C (2019) Expression of the potential therapeutic target claudin-18.2 is frequently decreased in gastric cancer: results from a large Caucasian cohort study. Virchows Arch 475:563–571. 10.1007/s00428-019-02624-7 31332522 10.1007/s00428-019-02624-7 PMC6861347

[20] Wang F, Yang Y, Du X, Zhu X, Hu Y, Lu C, Sui L, Zhao H, Song K, Yao Q (2023) Claudin18.2 as a potential therapeutic target for primary ovarian mucinous carcinomas and metastatic ovarian mucinous carcinomas from upper gastrointestinal primary tumours BMC Cancer 23:44. 10.1186/s12885-023-10533-x 10.1186/s12885-023-10533-x PMC9837907 36639622

[21] WHO WHO Classification of Tumours Editorial Board. Female genital tumours [Internet]. Lyon (France): International Agency for Research on Cancer; 2020 [cited 2023 April 26th]. (WHO classification of tumours series, 5th ed.; vol. 4). Available from: https://tumourclassification.iarc.who.int/chapters/34. Accessed April 26, 2023

[22] Prat J (2014) Staging classification for cancer of the ovary, fallopian tube, and peritoneum. Int J Gynaecol Obstet 124:1–5. 10.1016/j.ijgo.2013.10.001 24219974 10.1016/j.ijgo.2013.10.001

[23] Bierley J, Gospodarowicz MK, Wittekind C (eds) (2017) TNM classification of malignant tumours, 8th edn. Wiley Blackwell, Hoboken

[24] Sobin LHGM (2009) Wittekind C (2009) UICC TNM classification of malignant tumours (7th Edition). Wiley-Liss, New York

[25] Sobin LH, Wittekind C (2002) International Union Against Cancer (UICC) TNM classification of malignant tumours, 6th edn. UICC, New York

[26] Remmele W, Stegner HE (1987) Recommendation for uniform definition of an immunoreactive score (IRS) for immunohistochemical estrogen receptor detection (ER-ICA) in breast cancer tissue. Pathologe 8:138–140 3303008

[27] Vang R et al (2020) Mucinous carcinoma of the ovary. In: WHO Classification of Tumours Editorial Board. Female genital tumours [Internet], vol 4, 5th edn. WHO classification of tumours series. International Agency for Research on Cancer, Lyon. Available from: https://tumourclassification.iarc.who.int/chapters/34

[28] Muyldermans K, Moerman P, Amant F, Leunen K, Neven P, Vergote I (2013) Primary invasive mucinous ovarian carcinoma of the intestinal type: importance of the expansile versus infiltrative type in predicting recurrence and lymph node metastases. Eur J Cancer 49:1600–1608. 10.1016/j.ejca.2012.12.004 23321546 10.1016/j.ejca.2012.12.004

[29] Rodríguez IM, Prat J (2002) Mucinous tumors of the ovary: a clinicopathologic analysis of 75 borderline tumors (of intestinal type) and carcinomas. Am J Surg Pathol 26:139–152. 10.1097/00000478-200202000-00001 11812936 10.1097/00000478-200202000-00001

[30] Halimi SA, Maeda D, Shinozaki-Ushiku A, Koso T, Matsusaka K, Tanaka M, Arimoto T, Oda K, Kawana K, Yano T, Fujii T, Fukayama M (2013) Claudin-18 overexpression in intestinal-type mucinous borderline tumour of the ovary. Histopathol 63:534–544. 10.1111/his.12182 10.1111/his.12182 23905715

[31] Seidman JD, Khedmati F (2008) Exploring the histogenesis of ovarian mucinous and transitional cell (Brenner) neoplasms and their relationship with Walthard cell nests: a study of 120 tumors. Arch Pathol Lab Med 132:1753–1760. 10.5858/132.11.1753 18976011 10.5858/132.11.1753

[32] Seidman JD, Yemelyanova A, Zaino RJ, Kurman RJ (2011) The fallopian tube-peritoneal junction: a potential site of carcinogenesis. Int J Gynecol Pathol 30:4–11. 10.1097/PGP.0b013e3181f29d2a 21131840 10.1097/PGP.0b013e3181f29d2a

[33] Seidman JD, Horkayne-Szakaly I, Haiba M, Boice CR, Kurman RJ, Ronnett BM (2004) The histologic type and stage distribution of ovarian carcinomas of surface epithelial origin. Int J Gynecol Pathol 23:41–44. 10.1097/01.pgp.0000101080.35393.16 14668549 10.1097/01.pgp.0000101080.35393.16

[34] Peres LC, Cushing-Haugen KL, Köbel M, Harris HR, Berchuck A, Rossing MA, Schildkraut JM, Doherty JA (2019) Invasive epithelial ovarian cancer survival by histotype and disease stage. J Natl Cancer Inst 111:60–68. 10.1093/jnci/djy071 29718305 10.1093/jnci/djy071 PMC6335112

[35] Morice P, Gouy S, Leary A (2019) Mucinous ovarian carcinoma N Engl J Med 380:1256–1266. 10.1056/NEJMra1813254 30917260 10.1056/NEJMra1813254

[36] Türeci O, Sahin U, Schulze-Bergkamen H, Zvirbule Z, Lordick F, Koeberle D, Thuss-Patience P, Ettrich T, Arnold D, Bassermann F, Al-Batran SE, Wiechen K, Dhaene K, Maurus D, Gold M, Huber C, Krivoshik A, Arozullah A, Park JW, Schuler M (2019) A multicentre, phase IIa study of zolbetuximab as a single agent in patients with recurrent or refractory advanced adenocarcinoma of the stomach or lower oesophagus: the MONO study. Ann Oncol 30:1487–1495. 10.1093/annonc/mdz199 31240302 10.1093/annonc/mdz199 PMC6771222

[37] Morin PJ (2005) Claudin proteins in human cancer: promising new targets for diagnosis and therapy. Cancer Res 65:9603–9606. 10.1158/0008-5472.Can-05-2782 16266975 10.1158/0008-5472.Can-05-2782

[38] Hough CD, Sherman-Baust CA, Pizer ES, Montz FJ, Im DD, Rosenshein NB, Cho KR, Riggins GJ, Morin PJ (2000) Large-scale serial analysis of gene expression reveals genes differentially expressed in ovarian cancer. Cancer Res 60:6281–6287 11103784

[39] Agarwal R, D’Souza T, Morin PJ (2005) Claudin-3 and claudin-4 expression in ovarian epithelial cells enhances invasion and is associated with increased matrix metalloproteinase-2 activity. Cancer Res 65:7378–7385. 10.1158/0008-5472.Can-05-1036 16103090 10.1158/0008-5472.Can-05-1036

[40] Kleinberg L, Holth A, Trope CG, Reich R, Davidson B (2008) Claudin upregulation in ovarian carcinoma effusions is associated with poor survival. Hum Pathol 39:747–757. 10.1016/j.humpath.2007.10.002 18439941 10.1016/j.humpath.2007.10.002

[41] Yoshida H, Sumi T, Zhi X, Yasui T, Honda K, Ishiko O (2011) Claudin-4: a potential therapeutic target in chemotherapy-resistant ovarian cancer. Anticancer Res 31:1271–1277 21508375

[42] Choi YL, Kim J, Kwon MJ, Choi JS, Kim TJ, Bae DS, Koh SS, In YH, Park YW, Kim SH, Ahn G, Shin YK (2007) Expression profile of tight junction protein claudin 3 and claudin 4 in ovarian serous adenocarcinoma with prognostic correlation. Histol Histopathol 22:1185–1195. 10.14670/hh-22.1185 17647191 10.14670/hh-22.1185

[43] Sawada N (2013) Tight junction-related human diseases Pathol Int 63:1–12. 10.1111/pin.12021 23356220 10.1111/pin.12021 PMC7168075

[44] Gore M, Hackshaw A, Brady WE, Penson RT, Zaino R, McCluggage WG, Ganesan R, Wilkinson N, Perren T, Montes A, Summers J, Lord R, Dark G, Rustin G, Mackean M, Reed N, Kehoe S, Frumovitz M, Christensen H, Feeney A, Ledermann J, Gershenson DM (2019) An international, phase III randomized trial in patients with mucinous epithelial ovarian cancer (mEOC/GOG 0241) with long-term follow-up: and experience of conducting a clinical trial in a rare gynecological tumor. Gynecol Oncol 153:541–548. 10.1016/j.ygyno.2019.03.256 31005287 10.1016/j.ygyno.2019.03.256 PMC6559214

[45] https://oncologypro.esmo.org/meeting-resources/esmo-targeted-anticancer-therapies-congress/global-ring-study-determining-reproducibility-comparability-of-cldn18-testing-assays-in-gastric-cancer, Accessed on June 20, 2023

