# Supplementary material for: Spatial expression of claudin 18.2 in matched primaries and metastases of tubo-ovarian carcinoma of all subtypes
Source: Virchows Arch. 2024 Feb 7;485(1):63–74. doi: 10.1007/s00428-024-03756-1 (PMC11271439; doi:10.1007/s00428-024-03756-1)

**Virchows Archiv: Spatial expression of claudin 18.2 in matched primaries and metastases of tubo-ovarian carcinoma of all subtypes**

***Authors:***

- Paul Wagner 1*
- Paul Gass, MD 2
- Patrik Pöschke, MD 2
- Markus Eckstein, MD 1
- Laura Gloßner 1
- Arndt Hartmann, MD 1
- Matthias Wilhelm Beckmann, MD 2
- Peter Andreas Fasching, MD 2
- Matthias Ruebner, PhD 2
- Julius Emons, MD 2
- Ramona Erber, MD 1

*Affiliations:*

1 Institute of Pathology, University Hospital Erlangen, Friedrich-Alexander-Universität Erlangen-Nürnberg, Comprehensive Cancer Center Erlangen-EMN (CCC ER-EMN), Erlangen, Germany; paul.w.wagner@fau.de; ramona.erber@uk-erlangen.de; markus.eckstein@uk-erlangen.de; laura.glossner@fau.de; arndt.hartmann@uk-erlangen.de

2 Department of Gynecology and Obstetrics, University Hospital Erlangen, Friedrich-Alexander-Universität Erlangen-Nürnberg, Comprehensive Cancer Center Erlangen-EMN (CCC ER-EMN), Erlangen, Germany; paul.gass@uk-erlangen.de; patrik.poeschke@uk-erlangen.de; matthias.beckmann@uk-erlangen.de; matthias.ruebner@uk-erlangen.de; peter.fasching@uk-erlangen.de; julius.emons@uk-erlangen.de

**** Corresponding author:*** Paul Wagner; paul.w.wagner@fau.de; ORCID ID: 0009-0008-8257-656X

**Supplementary information**

**Supplemental Table S1** Detailed information on the number, size, and localization of the samples of tubo-ovarian carcinoma metastases in the tissue microarray

**Supplemental Fig. S1** Expression of CLDN18.2 in MTOC primaries: **A)** Overall positivity, **B)** IRS score mean with 95% CI, **C)** Distribution across IRS score groups, **D)** Intratumoral concordance between tumor centers and peripheries (Abbreviations: n, number; IRS, immunoreactive score; CI, confidence interval)

**Supplemental Fig. S2** Expression of CLDN18.2 in MTOC metastases: **A)** Overall positivity, **B)** IRS score mean with 95% CI, **C)** Distribution across IRS score groups, **D)** Intratumoral concordance between tumor centers and peripheries (Abbreviations: n, number; IRS, immunoreactive score; CI, confidence interval)

**Supplemental Fig. S3** Intertumoral concordance across the matched pairs of primary and metastasis of MTOC (**A**) and comparison of mean IRS scores between the two sites (**B**) (Abbreviations: n, number; IRS, immunoreactive score)

**Supplemental Fig. S4** Spatial immunohistochemical (IHC) CLDN18.2 expression patterns in mucinous tubo-ovarian carcinoma of the **A** expansile subtype and **B** infiltrative subtype (each letter from a-e symbolizing a single case; CLDN18.2 IHC, magnification x200, bar = 200 µm)

**Supplemental Fig. S5** Spatial immunohistochemical (IHC) CLDN18.2 expression patterns in metastatic mucinous tubo-ovarian carcinoma with a predominantly infiltrative pattern (each letter from a-e symbolizing a single case, magnification x200, bar = 200 µm)

**Supplemental Table S1** Detailed information on the number, size, and localization of the samples of tubo-ovarian carcinoma metastases in the tissue microarray

| | **Peritoneal** | | **Organ parenchyma (T status relevant)** | | **Regional lymph node** | | **Distant metastases** | |
|---|---|---|---|---|---|---|---|---|
| **Number** | 384 | | 58 | | 17 | | 19 | |
| **Relapses** | 17 | | 7 | | 4 | | 9 | |
| **Size** | | | | | | | | |
| | *< 2 cm* | 192 | *< 2 cm* | 5 | *< 1 cm* | 6 | *< 2 cm* | 10 |
| | *> 2 cm* | 192 | *> 2 cm* | 53 | *> 1 cm* | 11 | *> 2 cm* | 9 |
| **Localization** | | | | | | | | |
| | *Inside pelvis* | 126 | *Colon* | 9 | *Intra-peritoneal* | 1 | *Liver* | 4 |
| | *Outside pelvis* | 258 | *Sigma* | 18 | *Paraaortal* | 13 | *Skin* | 7 |
| | | | *Rectum* | 10 | *Pelvin* | 3 | *Lymph node* | 4 |
| | | | *Small intestine* | 4 | | | *Lung/ Pleura* | 2 |
| | | | *Stomach* | 2 | | | *Mamma* | 1 |
| | | | *Uterus* | *13* | | | *Vagina* | 1 |
| | | | *Vagina* | 2 | | | | |

**Supplemental Fig. S1** Expression of CLDN18.2 in MTOC primaries: **A)** Overall positivity, **B)** IRS score mean with 95% CI, **C)** Distribution across IRS score groups, **D)** Intratumoral concordance between tumor centers and peripheries (Abbreviations: n, number; IRS, immunoreactive score; CI, confidence interval)

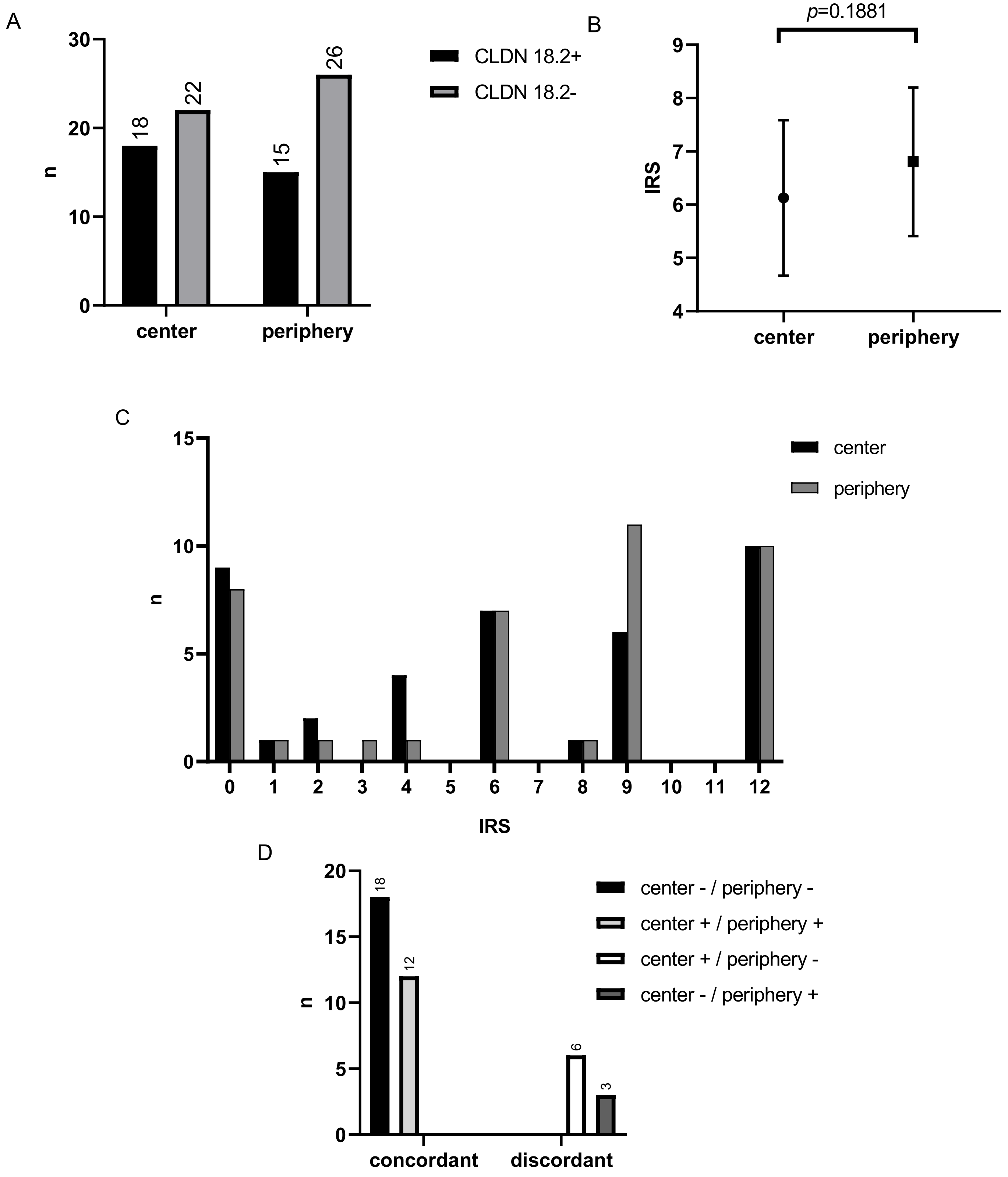

**Supplemental Fig. S2** Expression of CLDN18.2 in MTOC metastases: **A)** Overall positivity, **B)** IRS score mean with 95% CI, **C)** Distribution across IRS score groups, **D)** Intratumoral concordance between tumor centers and peripheries (Abbreviations: n, number; IRS, immunoreactive score; CI, confidence interval)

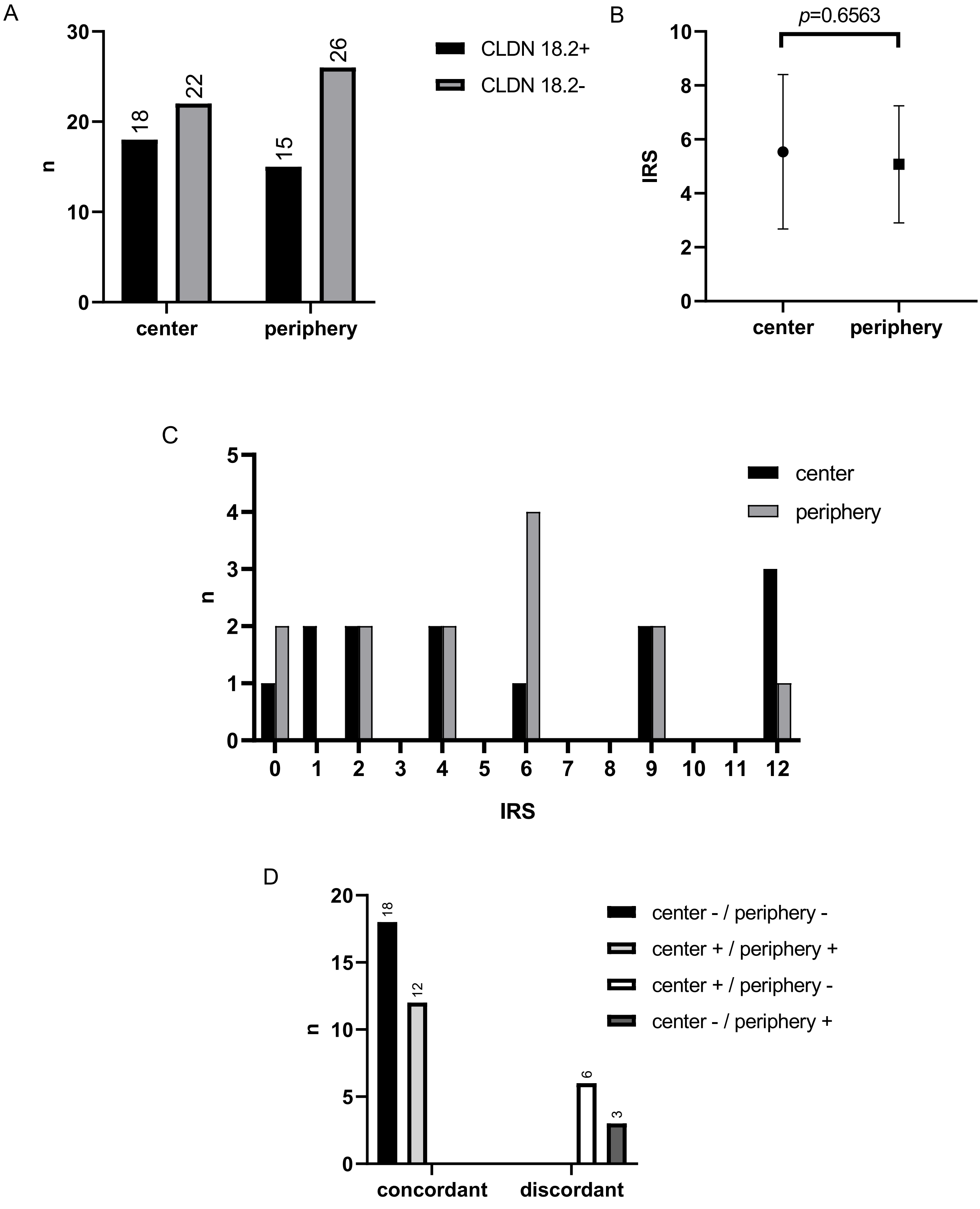

**Supplemental Fig. S3** Intertumoral concordance across the matched pairs of primary and metastasis of MTOC (**A**) and comparison of mean IRS scores between the two sites (**B**) (Abbreviations: n, number; IRS, immunoreactive score)

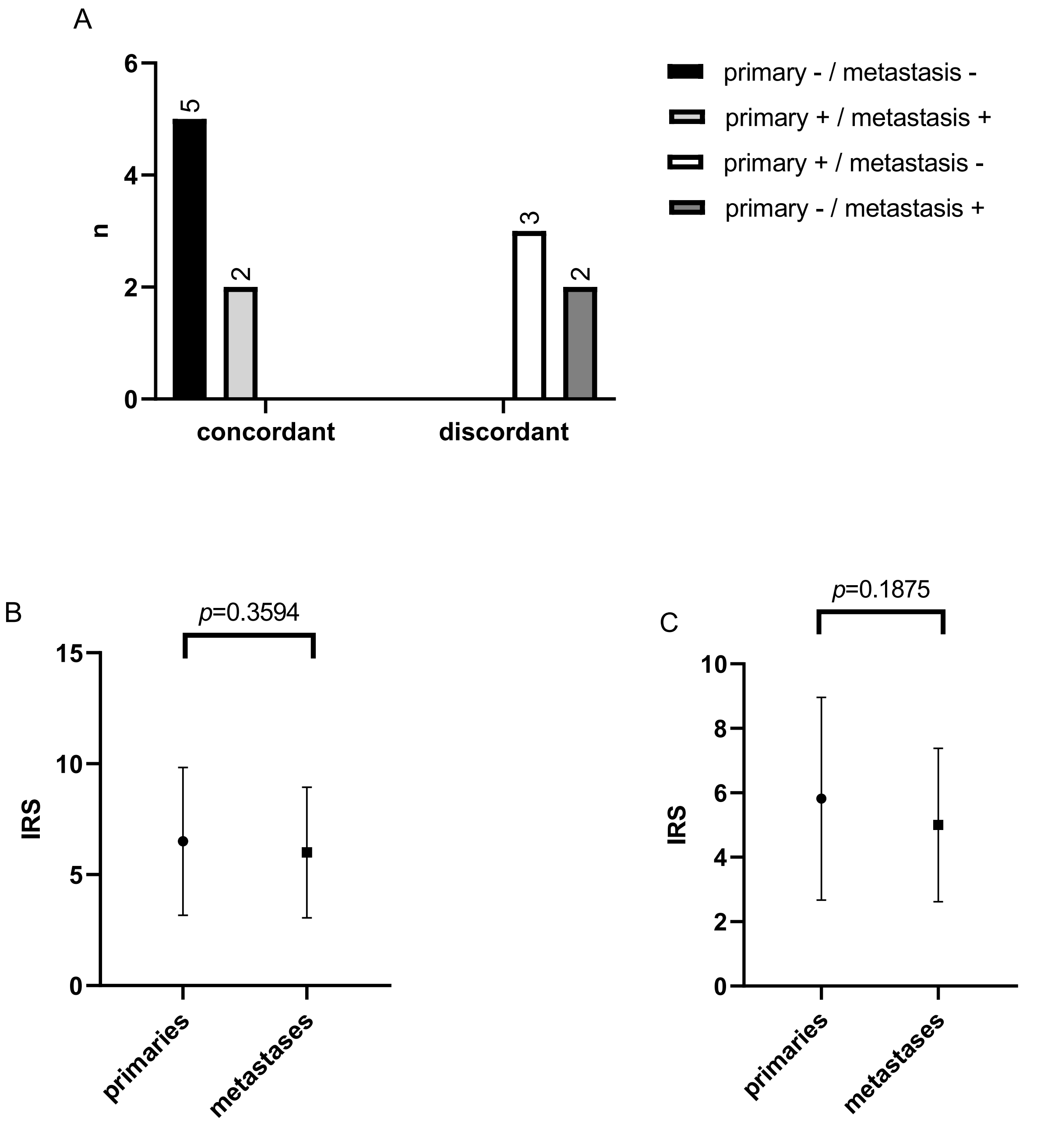

**Supplemental Fig. S4** Spatial immunohistochemical (IHC) CLDN18.2 expression patterns in mucinous tubo-ovarian carcinoma of the **A** expansile subtype and **B** infiltrative subtype (each letter from a-e symbolizing a single case; CL DN18.2 IHC, magnification x200, bar = 200 µm)

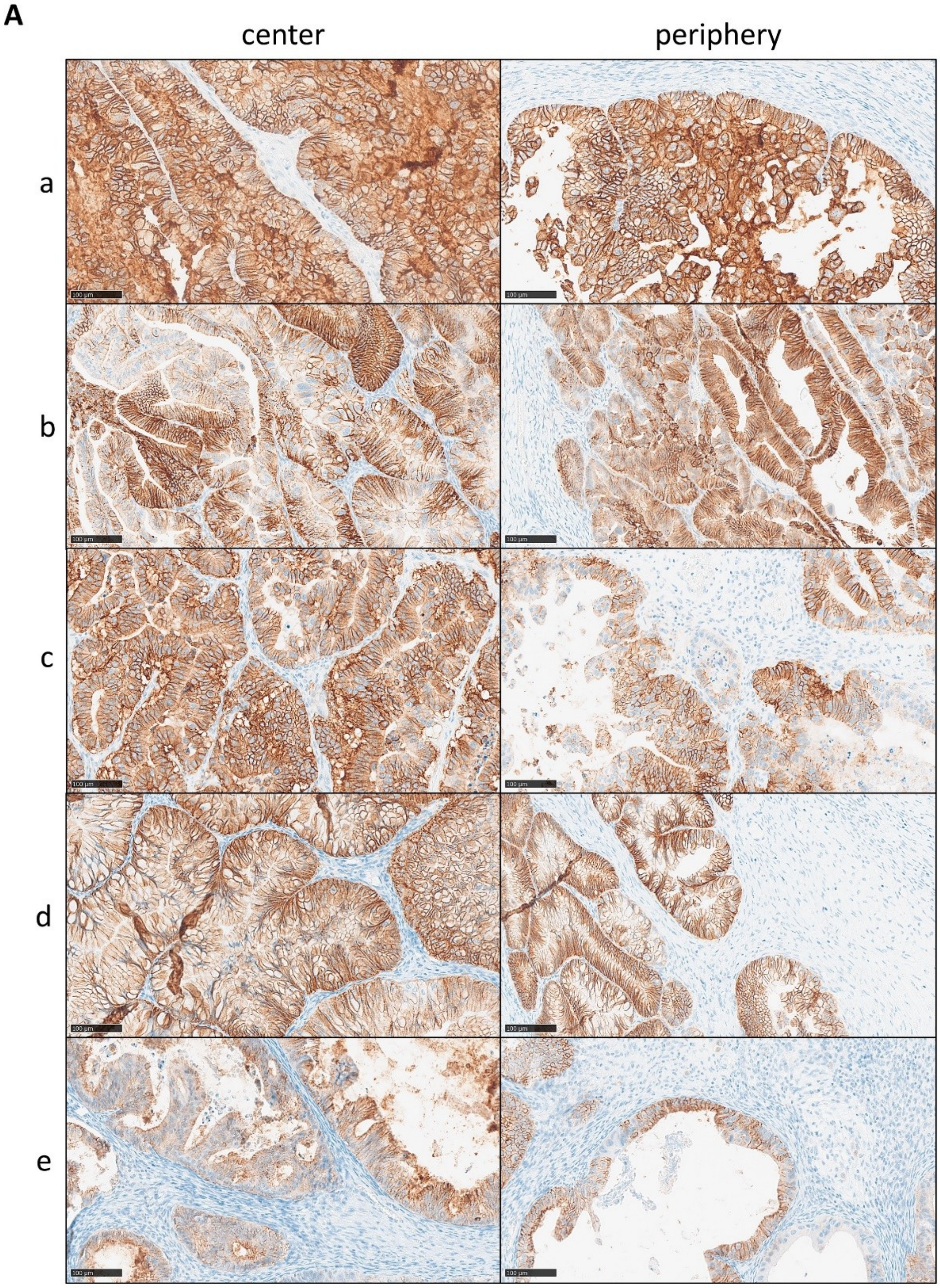

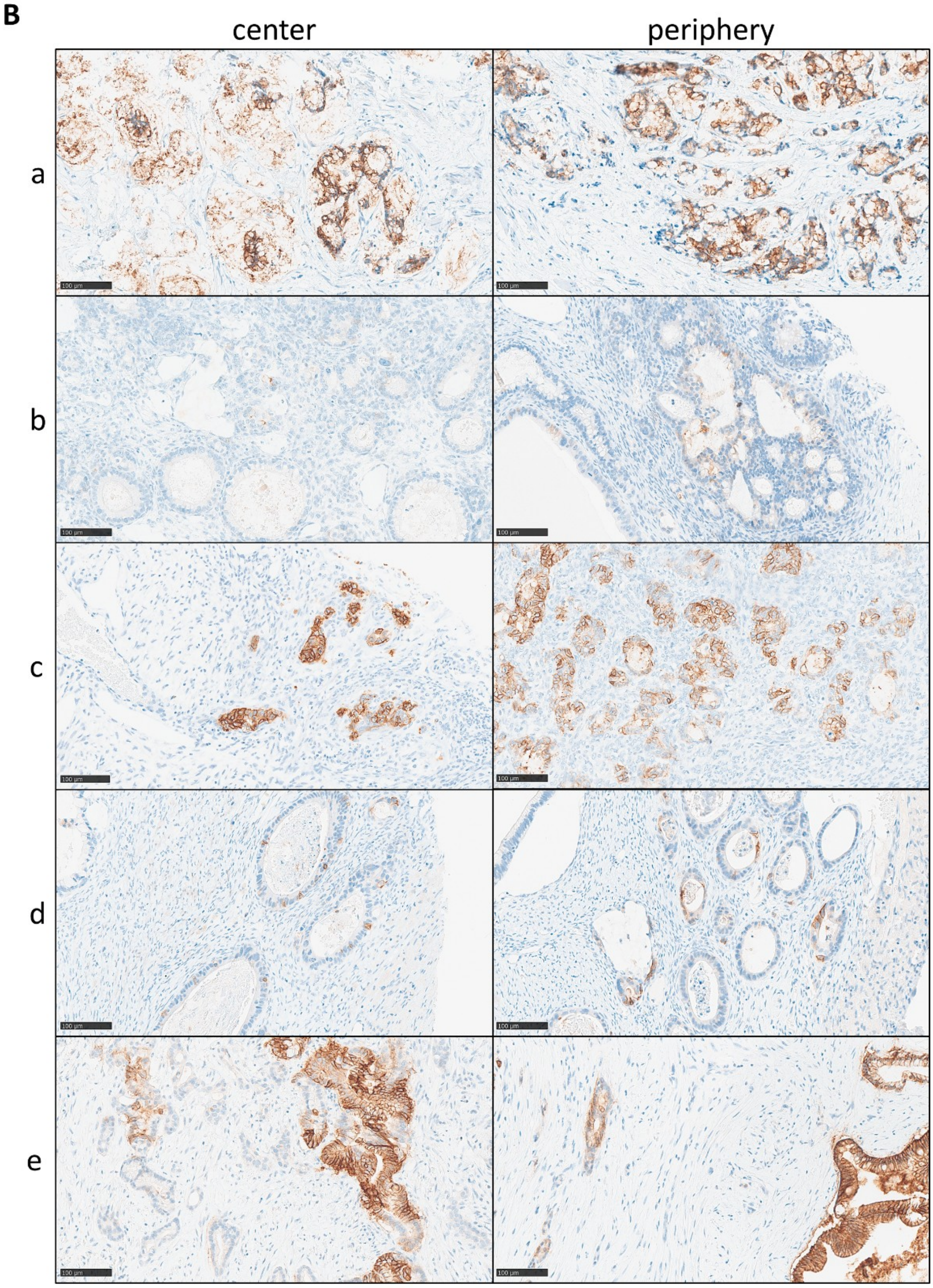
B
center
periphery
a
b
c
d
e

**Supplemental Fig. S5** Spatial immunohistochemical (IHC) CLDN18.2 expression patterns in metastatic mucinous tubo-ovarian carcinoma with a predominantly infiltrative pattern (each letter from a-e symbolizing a single case, magnification x200, bar = 100 µm)

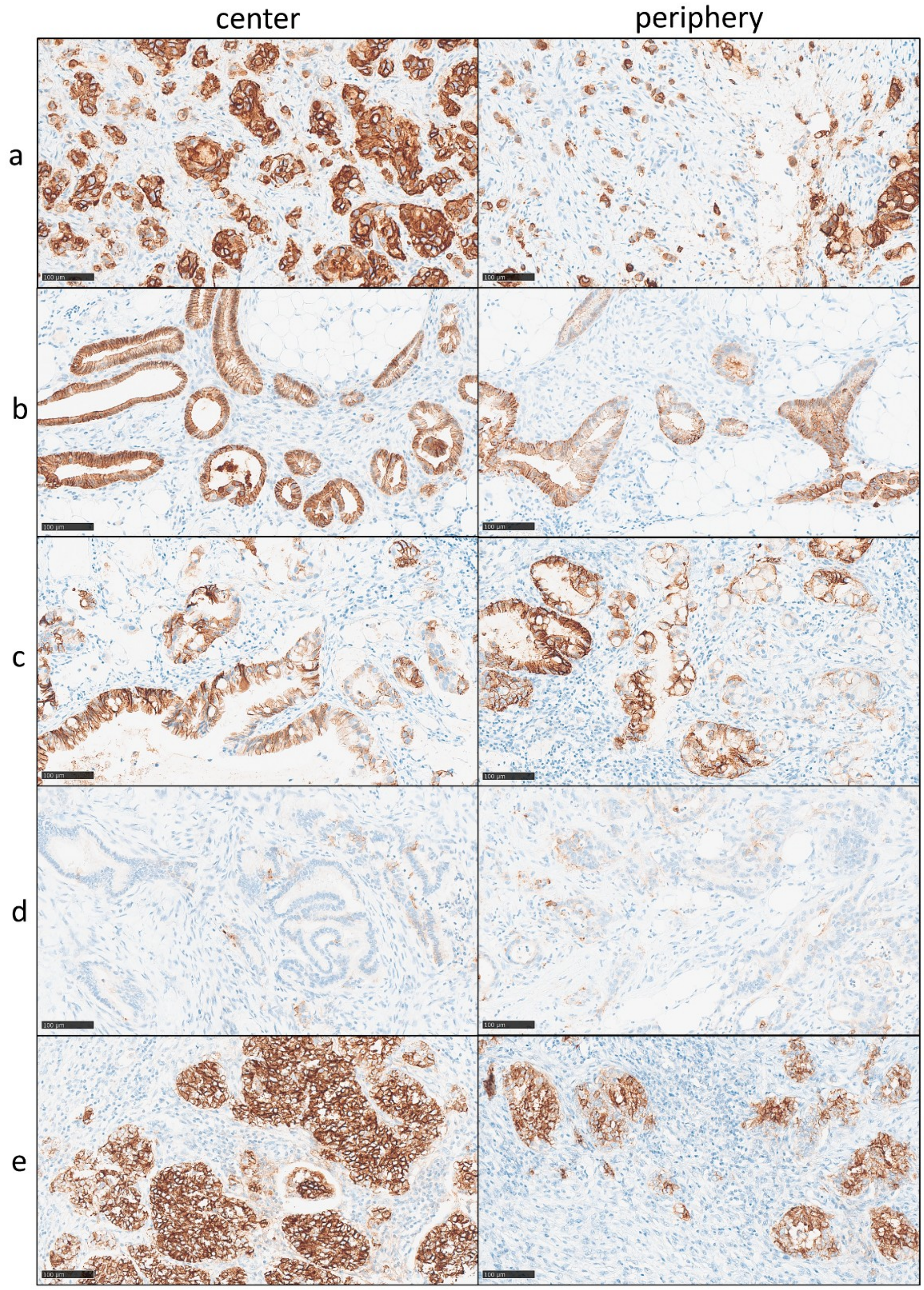

Supplement: Supplementary file 1 — Supplementary file1 (PDF 2513 KB) [file 428_2024_3756_MOESM1_ESM.pdf]
